# Migration and health: a global public health research priority

**DOI:** 10.1186/s12889-018-5932-5

**Published:** 2018-08-08

**Authors:** Kolitha Wickramage, Jo Vearey, Anthony B. Zwi, Courtland Robinson, Michael Knipper

**Affiliations:** 1 Migration Health Division, International Organization for Migration, United Nations Migration Agency, Geneva, Switzerland; 2 African Centre for Migration & Society, University of the Witwatersrand and Centre of African Studies, University of Edinburgh, PO Box 76, Wits, 2050 South Africa; 3 Health, Rights and Development (HEARD@UNSW), School of Social Science, The University of New South Wales, Sydney, NSW 2052 Australia; 4 Center for Humanitarian Health, Johns Hopkins Bloomberg School of Public Health, Baltimore, MD USA; 5 Institute of the History of Medicine, University Justus Liebig Giessen, Iheringstr. 6, 35392 Giessen, Germany

**Keywords:** Migration, Health, Global health, Research

## Abstract

**Background:**

With 244 million international migrants, and significantly more people moving within their country of birth, there is an urgent need to engage with migration at all levels in order to support progress towards global health and development targets. In response to this, the 2nd Global Consultation on Migration and Health– held in Colombo, Sri Lanka in February 2017 – facilitated discussions concerning the role of research in supporting evidence-informed health responses that engage with migration.

**Conclusions:**

Drawing on discussions with policy makers, research scholars, civil society, and United Nations agencies held in Colombo, we emphasize the urgent need for quality research on international and domestic (in-country) migration and health to support efforts to achieve the Sustainable Development Goals (SDGs). The SDGs aim to ‘leave no-one behind’ irrespective of their legal status. An ethically sound human rights approach to research that involves engagement across multiple disciplines is required. Researchers need to be sensitive when designing and disseminating research findings as data on migration and health may be misused, both at an individual and population level. We emphasize the importance of creating an ‘enabling environment’ for migration and health research at national, regional and global levels, and call for the development of meaningful linkages – such as through research reference groups – to support evidence-informed inter-sectoral policy and priority setting processes.

## Background

Migration and health are increasingly recognized as a global public health priority [1]. Incorporating mixed flows of economic, forced, and irregular migration, migration has increased in extent and complexity. Globally, it is estimated that there are 244 million international migrants and significantly more internal migrants – people moving within their country of birth [2]. Whilst the majority of international migrants move between countries of the ‘global south’ [2], these movements between low and middle-income countries remain a “blind spot” for policymakers, researchers and the media, with disproportionate political and policy attention focused on irregular migration to high-income countries. Migration is increasingly recognized as a determinant of health [3–5]. However, the bidirectional relationship between migration and health remains poorly understood, and action on migration and health remains limited, negatively impacting not only those who migrate but also sending, receiving, and ‘left-behind’ communities [1].

In February 2017, an international group of researchers participated in the 2nd Global Consultation on Migration and Health held in Colombo, Sri Lanka with the objectives of sharing lessons learned, good practices, and research in addressing the relationship between migration and health [1]. Hosted by the International Organization for Migration (IOM), the World Health Organization (WHO), and the Sri Lankan government, the Global Consultation brought together governments, civil society, international organizations, and academic representatives in order to address migration and health. The Consultation facilitated engagement with the health needs of migrants, reconciling the focus on long-term economic and structural migration - both within and across international borders - with that of acute, large-scale displacement flows that may include refugees, asylum seekers, internally displaced persons and undocumented migrants.

The Consultation was organised around inputs on three thematic areas: Global Health [6]; Vulnerability and Resilience [7]; and, Development [8]. These inputs guided working group discussions exploring either policy, research, or monitoring in relation to migration and health. This paper reports on the outcomes of the research group after an extensive period of debate at the Consultation and over the subsequent 9 months. We identify key issues that should guide research practice in the field of migration and health, and outline strategies to support the development of evidence-informed policies and practices at global, regional, national, and local levels [9]. Debate and discussion at the Consultation, and below, were guided by two key questions:

- What are the opportunities and challenges, and the essential components associated with developing a research agenda on migration and health?
- What values and approaches should guide the development of a national research agenda and data collection system on migration and health?

Our discussions emphasized that international targets, such as the Sustainable Development Goals (SDGs) and Universal Health Coverage (UHC; Health target 3.8 of the SDGs), are unlikely to be achieved if the dynamics of migration are not better understood and incorporated in policy and programming. To address this, and in order to improve policy and programming, a renewed focus on enhancing our understanding of the linkages between both international and internal migration and health, as well as the outcomes and impacts arising from them, is urgently needed.

## Main text

### Migration and health research: Leave no-one behind

The Sustainable Development Goals (SDGs) identify migration as both a catalyst and a driver for sustainable development. A clarion call of the SDGs is to ‘leave no-one behind’, irrespective of their legal status, in order to achieve Universal Health Coverage (UHC) for all [10]. In many countries, however, equitable access to health services is considered as a goal only in relation to citizens. Additionally, internal migration is left out of programming and policy interventions designed to support UHC for all. While UHC aims at ensuring “everyone” can access affordable health systems without increasing the risk of financial ruin or impoverishment, the formulation of UHC remains unclear regarding non-nationals/non-citizens [11]. While many international declarations state that the right to health applies to all, including migrants and non-citizens, many national policies exclude these groups in whole or part [12].

In addition to international and internal migration, the health concerns associated with labour migration require attention; migrant workers are estimated to account for 150.3 million of the 244 million international migrants [2]. While labour migration leads to significant economic gains for countries of origin and destination, true developmental benefits are only realised with access to safe, orderly and humane migration practice [13]. Many migrant labourers work in conditions of precarious employment, within ‘difficult, degrading and dangerous’ jobs yet little is known about the health status, health outcomes, and resilience/vulnerability trajectories of these migrant workers and their ‘left behind’ families. Many undergo health assessments as a pre-condition for travel and migration, yet many such programs remain unlinked to national public health systems [14].

**Fig. 1:**
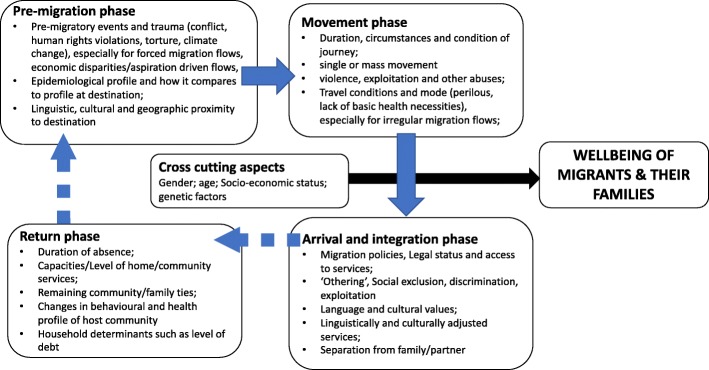
Factors influencing health and wellbeing of migrants and their families along the phases of migration

**Fig. 2:**
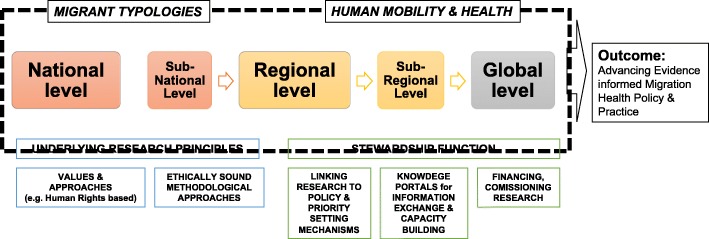
Advancing Migration and Health Research at National, Regional and Global Levels: a conceptual framework

Our discussions highlighted the complex and heterogeneous nature of research on migration and health, with particular concerns raised around the emphasis on international rather than internal migration, in view of the greater volume of the latter. The need for a multilevel research agenda to guide appropriate action on international and internal migration, health, and development was highlighted. In order to account for immediate, long-term and inter-generational impacts on health outcomes, migration and health research should: (1) incorporate the different phases of migration (Fig. 1); (2) adopt a life-course approach; and, (3) integrate a social determinants of health (SDH) approach.

Unease was expressed about the increasingly polarised political viewpoints on migration, often propagated by nationalist and populist movements, which present real challenges to researchers. This may also be associated with a reluctance to finance research exploring discriminatory policies that limit the access of international migrants to health services and other positive determinants of health, including work and housing.

The increasing complexity of global, regional, and national migration trends, as well as disagreements about the correct way to define and label different types of migrants, create additional difficulties within an already tense and politically contested research domain. Associated with this are the particular challenges associated with collecting and utilising data on ‘irregular migrants’ – international migrants currently without the documentation required to legally be in a particular country. These undocumented migrants, often living in the shadows of society, are more vulnerable to poor health outcomes due to restrictive policies on access to health and social services, to safe working and living conditions, and/or a reluctance to access services for fear of arrest, detention and/or deportation [15, 16]. Whilst arguments for improving access to health care for marginalised migrants are based on principles of equity, public health, and human rights, the importance of research on the economic implications of limiting access to care for international migrants was highlighted [2]. This challenging terrain generated a myriad of research questions during the group discussions (Table 1).

**Table 1:** Examples of research questions at the nexus of migration, health and development to drive policy and practice

• *National*: What are the experiences of migrants in accessing health care systems? How do these differ along migration trajectories/journeys, and by migrant typology, by age of migrant, by gender, by country of origin? What are their beliefs, understandings, values and health literacy? How (and in what direction) does health vulnerability and resiliency change across the four phases of the migration cycle (pre-departure, during transit, at destination and upon return)?	
• *National*: Beyond Member States’ obligations under international human rights law, does providing access to regular primary health care services including preventive services such as immunization for migrants in an irregular situation (rather than only allowing them access to select emergency healthcare) be cost-saving for national healthcare systems? What are the short- and long-term economic effects of restrictive versus integrative approaches/policies?	
• *National/Regional*: Does "low-skilled" labour migration (especially from low-income countries) cause negative health and social consequences to those ‘left-behind’ migrant households? Or, do such migrants and their families thrive by use of remittances to purchase better food, health care and education? Do such risks/rewards change over time? What interventions are effective in reducing health vulnerabilities of such migrant families?	
• *Regional/Global*: What role does human mobility play in globalization of health risks, and for the reintroduction of diseases such as Malaria in elimination or near elimination settings? To what extent have countries enshrined the right to health for migrant populations within preparedness and response plans for disease outbreaks or other public health events?	

### Towards a framework for advancing migration and health research

The consultation took into account the extensive research experience of the group (see Appendix), as well as engagement with key literature and context-specific evidence [see, for example 1–7]. Discussion led to the development of a framework that brings together what we identify as the key components for advancing a global, multi-level, migration and health research agenda (Fig. 2). Two areas of focus to advance the migration and health research agenda were identified: (1) *exploring health issues across various migrant typologies*, and (2) *improving our understanding of the interactions between migration and health*. Advancing research in both areas is essential if we are to improve our understanding of how to respond to the complex linkages between both international and internal migration and health. This, we argue, can be achieved by moving away from an approach that exceptionalises migration and migrants, to one that integrates migration into overall health systems research, design, and delivery, and conceptualises this as a way to support the achievement of good health for all.

Building from these focus areas, our framework outlines the essential components for the development and application of multi-level research on migration and health. First are key principles underlying research practice: promoting interdisciplinary, human rights oriented, ethically sound approaches for working with migrants. Second are multi-level stewardship functions needed to meaningfully link migration and health research to policy practice and priority setting, [17]. This includes establishing knowledge exchange mechanisms, financing, commissioning, and utilising research to guide evidence informed policies. This may better enable health systems to become ‘migration aware’ [18] or what the International Organization for Migration (IOM) terms ‘mobility competent’ - sensitive to health and migration [1].

#### Migration and health research: Two key focus areas

##### Migrant typologies

To assist in understanding the associations between migration and health, our research must find ways to better capture and engage with complex, dynamic, and often intersecting migrant typologies. We must be careful not to cluster migrants and their associated lived experiences, to simple, reductionist categories such as internal versus cross-border or documented versus undocumented, or even refugee versus economic migrant [19]. However, we do need a way of categorising different migrant groups when, for example, exploring epidemiological profiles and associated burdens of disease. To do this, we need to develop a set of nuanced yet flexible typologies that are able to capture the contextually relevant factors affecting migrant experiences, at both the individual and population levels. As outlined in Table 2, this will require careful consideration of multiple factors to assist us in improving our understandings of the ways in which diverse migrant groups are associated, or not, with various health and wellbeing outcomes. Definitions that are based on immigration status - such as ‘refugee’, ‘immigrant’ or ‘asylum seeker’ - will incorporate diverse sub-groups, often with different levels of health vulnerabilities and resiliencies based on their migration trajectory. For instance, a refugee entering a country with an offer of permanent resettlement or with a recognized temporary protected status, will have different opportunities and challenges than an asylum-seeker, or migrant worker, crossing a border possibly without documents or a clear pathway to needed healthcare and protections. Each of these migrating populations carry different health burdens (and resiliencies) from their country of origin, their social position and access to resources, and their migration experiences; and each will face different barriers and uncertainties as they seek access to services, support and integrate in host communities. The definitions of migrant groups adopted by states not only need clear elucidation but also need to reflect the context-specific conditions affecting health access and protection. In Europe, for example, the entitlements to health care for asylum seekers differ by country [20]. The Migrant Integration Policy Index (MIPEX) health strand was developed as a tool to monitor policies affecting migrant integration in 38 different countries [18]. It measures the equitability of policies relating to four issues: migrants’ entitlements to health services; accessibility of health services for migrants; responsiveness to migrants’ needs; and measures to achieve change. Such tools are important steps in assessing migrant integration and for implementing migrant-sensitive policies that are aligned with the person-centred UHC principles.

**Table 2:** Examples of the factors/variables involved in the development migrant typologies

• *Migrant status*: nationality; documentation status; tourists; business- travellers; job seeker; refugees; irregular migrants; asylum seekers; internally displaced persons; migrant workers	
• *Geography*: rural to urban migrants; intra-urban migration; inter-regional migration; internal migration; transnational migration; return migration	
• *Temporality*: weekly/monthly commuting; seasonal migration; labour related contractual migration; short-term or protracted migration; time in transit	
• *Socio-demographic status*: age; gender; family structure; economic status; education level; level of professional and occupational skills	
• *Motivations/Causal classifications*: job seeking; family reunification; asylum seeking; refugee resettlement; labour migration; student migration	

An awareness of this complexity underlies the need to document multiple migrant voices and migration experiences along the diverse trajectories when exploring associations between migration and health. This could, for instance, involve capturing the voices of children and other family members ‘left-behind’ as a result of labour migration, or of seasonal migrant workers. Research into the issues, policies and programmes that influence health and health literacy among migrant populations and the role that communities, households, industries, schools, and transnational networks play in promoting health also needs exploration.

Key challenges exist when attempting to use and compare migration data internationally, as a result of differences in the definition of who is an international migrant, non-national, or internal migrant; inconsistent data sources; and limited data coverage. A recent analysis of the availability, reliability and comparability of data on international migration flows in European countries noted that “comparing migration flows in various countries would be like comparing pears and apples” [21]. The use of standard indicators can result in unreliable data if migration dynamics are not considered. For example, measures of life expectancy are skewed if international migrants return to their home countries when they are seriously ill, but their departure is not accounted for in vital registration or other systems [21]. Reporting that is based on incomplete, poor quality or non-comparable population data that fails to measure and/or report migration can give rise to misleading conclusions and limits the validity of data interpretation.

#### Research at the nexus of migration and health

We recognise the bi-directionality of the relationship between migration and health. Our research should explore how different forms of migration influence health – at both individual and population levels - and how health status affects decisions to migrate and shapes post-migration experience. Migration trajectories can positively or negatively impact health outcomes, just as health status can affect migration outcomes; this two-way relationship should be better reflected in research. To support this, we must be sure to differentiate carefully between different migrant typologies – for example within or across international borders and for what purpose: work, family reunification, escape from persecution, flight from conflict or natural disaster, or to seek asylum. Each of these operates within substantially different contexts whether one takes the migrant and their health into account, or their rights and entitlements, or how they are seen by the dominant society or community to which they migrate. We recognise that being a migrant is not in itself a risk to health: it is the conditions associated with migration that may increase vulnerability to poor health [4]. Owing to the ways in which people move and the spaces they traverse or at which they arrive, migrants may reside in - or pass through - ‘spaces of vulnerability’ [22] – key spaces associated with potentially negative health outcomes – including along transport corridors, urban slums, construction sites, commercial farms, fishing communities, mines, and detention centres. Such spaces may contain a combination of social, economic and physical conditions that may increase the likelihood of exposure to violence and abuse and/or acquisition of communicable or non-communicable disease [22]. The daily stressors that may be experienced in these spaces are increasingly acknowledged to affect emotional wellbeing and mental health [23].

As migration is an ever-changing dynamic process, generating and maintaining timely and comparable migration data and improving relevant information systems is important. ‘Quick wins’ in obtaining migration and health data by integrating migration variables into existing national demographic and health surveys, for instance, were highlighted. National disease control programs such as tuberculosis, HIV and malaria control programs should also be encouraged to collect data on internal and international migration, especially in cross-border areas. Communicable disease control remains a key health concern associated with human migration. Our discussions recognised the importance of embracing systems-theory approach for improving understanding of how migration influences not only disease transmission but also health promotion, and health-care seeking behaviours. The importance of collecting such data with strict adherence to research ethics and human rights was emphasised.

## Conclusions

### Towards a multilevel migration and health research agenda

To effectively inform policies and programs on migration and health, it is essential to invest in evidence generation through research at local, national, regional, and global levels. Identified approaches include the establishment of research reference groups at each level to support, guide, and connect the development and application of research to support evidence-informed policy making at multiple levels. Mapping and analysis of key stakeholders, migration patterns, existing legal frameworks, data source, and research output via bibliometric analysis is needed. Multi-level migration and health policy and priority setting processes must be guided by interdisciplinary and multisectoral thinking in order to address the multiple determinants associated with the health of both internal and cross-border migrants.

Key constituencies need to be mobilised from academia, civil society, international organizations, the private sector including employer groups, trade unions and migrant worker networks. These groups may also play a role in commissioning or directly undertaking applied research in order to advance better outcomes for migrants and communities in both places of origin and destination. High-level political leadership and health and development champions should raise the visibility of migration and health research. It is important to utilise existing research structures and resources to support the development of a research agenda on migration and health, as well as to seek support for the development of dedicated research commissions on migration and health at multiple levels in order to harness evidence to drive policy-making and programme formation. For instance, the Government of Sri Lanka, with the technical cooperation of IOM, commissioned a National Migration Health Research Study in 2010 to explore health impacts of inbound, outbound, and internal migrant flows including those of left-behind migrant families. The research findings ultimately contributed to the formulation of an evidence-informed National Migration Health Policy and national action plan in 2013 [24]. The research was led through local research institutions and research process were linked to an inter-ministerial and inter-agency process chaired by the Minister of Health. This evidence informed policy making process also led to a number of national programs such as ‘the national border health program’ in 2013, revitalizing domestic legal frameworks on health security, and advancing health protection of migrant workers at regional inter-governmental initiatives such as the Colombo Process.

#### Regional

At the regional level, consultative processes are required to develop common approaches to migration and health, including communicable disease surveillance, monitoring of interventions, applied research collaboration across national borders and capacity building – particularly interdisciplinary postgraduate training. For instance, the Mekong Basin Disease Surveillance (MBDS) Consortium is a sub-regional co-operation spearheaded by health ministries from member countries Cambodia, China, Lao PDR, Myanmar, Thailand and Vietnam [25]. In relation to labour migration, regional processes – such as the *Colombo Process* [26] - should explore the management of overseas employment and contractual labour. In addition, migrant health-related concerns should be emphasised in the negotiation of free trade agreements that increase migration between states, such as the Post-2015 Health Development Agenda for a “*Healthy, Caring and Sustainable Community*” initiative of the Association of South-East Asian Nations (ASEAN) [27] and efforts to implement the “Health in all Policies” strategy of the European Union [7].

#### Global

Methods to map human mobility for public health preparedness and response stemming from outbreaks and other health emergencies are needed in order to provide accurate information on population movements, for monitoring the progression of outbreaks, predicting future spread and allocating resources for surveillance and containment strategies. Human mobility was a critical factor in the spread of Ebola virus in the West African region.

A coordinated global research agenda on migration and health is urgently needed. Potential elements include collaboration with stakeholders involved in implementing global initiatives – such as the SDGs – to ensure that indicators and data collection strategies are sensitive to both internal and cross-border migration, and health related issues. Identification of datasets and data collection processes that can be adapted and mined for disaggregated health data related to migration are also crucial in advancing the evidence base. We support the development of a sustainable global reference group that can share research evidence, expertise and experience, develop methodological and ethical guidelines, undertake multi-country studies, provide training and build a global knowledge hub in migration and health. Such a group can also mobilise funders and development partners, collaborate with scientific and professional associations, and engage with journals and publishers to create awareness on the need to better promote migration and health research.

The *‘Migration, Health, and Development Research Initiative’* (MHADRI) is a global network of academics and other research partners who aim to advance migration and health research practice [28]. The research network was formed around the need to build a global alliance of migration and health researchers and provide a platform to share, collaborate, develop, mentor, advocate and disseminate inter-disciplinary research at the nexus of health and migration. A key goal of the network is to enable researchers from developing nations the opportunity to collaborate and promote research in the Global South. The network has grown to encompass 100 researchers globally, across diverse disciplines, geographic areas and stages of career. A global reference group would be well placed to develop good practice guides on data collection systems, research methods and ethics; research translation and dissemination; and, policy integration strategies.

### Research principles

We identified core principles that should guide research on migration and health, and work with migrant populations: an ethically sound human rights approach to research that involves engagement across multiple disciplines. Researchers need to be sensitive when designing and disseminating research findings as data on migration and health may be misused, both at an individual and population level. Key questions related to how researchers can exercise their duty of care as they engage in research, and how we can promote careful use of data and research to make sure it does more good than harm. Activities associated with international migration sometimes take place in a climate of victim blaming, othering, and stigmatisation that prioritises purported national security concerns [29]. Pressing concerns were identified that relate to the ways in which researchers can navigate this increasingly challenging environment, and how trust can be established among different stakeholders – including with international migrant groups. Securitization agendas also affect the health of migrants by excluding, discriminating and/or blaming migrants as vectors of disease. Ethical approaches to research, with a clear commitment to universal human rights, are therefore paramount in a climate of increasingly restrictive immigration regimes.

Discussions also highlighted the challenges associated with the collection of data with and from migrant populations. These include sampling, biases, and practical barriers such as language and culture, as well as the challenges inherent in reaching people who are often highly marginalised and potentially criminalised. Particular attention needs to be given to ethical issues: protecting confidentiality and ensuring that participation in research does not have an adverse impact on migrants, especially irregular migrants, and that participants gain access to relevant services if required. The development of meaningful partnerships and respectful research practice with actors involved in the migration process will also improve the quality, reliability, legitimacy, and use of the data generated.

Contributions from a range of disciplines – such as anthropology, demography, sociology, law, political science, psychology, policy analysis, public health, and epidemiology – are required to unpack the complex relationships between migration and health. Approaches to “slow research” [30] may help increase the sensitivity of epistemologies and methods to local realities, intricate dynamics, and the multiple voices and perceptions of migrants, health professionals and other individuals involved [24]. However, the lack of dedicated research units, institutes or centres on migration and health - especially within lower-income country contexts - require existing researchers and scholars to consolidate and better engage with sub-regional, regional and global research networks to ensure capacity building, mentoring, and support. Sensitising the donor community to the migration and health agenda, especially those funding research, is paramount. Curriculum development and teaching support for building the next generation of migration and health researchers is critical to successfully building and sustaining future research on migration and health.

### Stewardship elements

We discussed the importance of developing appropriate research translation and engagement activities in order to support key, identified stewardship functions [17] at the global, regional, national and local levels. Key gaps in stewardship related to the lack of major funding mechanisms for research at national, regional, and global levels, and the need to invest in capacity building for emerging researchers through training programs and support, especially for researchers in lower-income country settings. Collaboration is required to support relationships among researchers and with relevant stakeholders, particularly with migrant communities. This includes building inclusive migration and health research networks, developing communities of practice, and supporting collaborations with those working on other global health priorities. Our research also needs to include the experiences of service providers who engage with various migrant populations, such as those within the health care sectors, border management, law enforcement, and labour migration. The development of effective research translation and public engagement strategies for sharing research findings is critical: not only to shape multi-level policy processes but also public and political opinion.

There was clear consensus on our commitment to enhancing the quality and breadth of multi-level research evidence to support the development of improved responses to migration and health. The importance of an ‘enabling environment’ for migration and health research at local, national, regional and global levels was emphasised, as was the development of meaningful linkages – such as through research reference groups – to support evidence-informed and intersectoral policy and priority setting processes. Our research needs to be underpinned by a human rights approach to health and sound ethical practice. With adequate funding, capacity development, and support for academic freedom, we can improve the evidence base to guide policy and programming for migration and health at multiple levels and in so doing contribute to improving health for all.

## Appendix

### Members of the research stream at the 2nd Global Consultation on Migration and Health

In alphabetical order:

1. Ibrahim Abubakar (Director, Institute for Global Health, University College London, United Kingdom)
2. Anjali Borhade (Associate Professor, Indian Institute of Public Health, India)
3. Chee-khoon Chan (Research Associate, University of Malaya, Malaysia)
4. Julia Puebla Fortier (Executive Director, Diversity Rx - Resources for Cross Cultural Health Care)
5. Charles Hui (Associate Professor of Paediatrics and Chief of Infectious Diseases, University of Ottawa, Ottawa, Ontario)
6. Michael Knipper (Associate Professor, Institute of the History of Medicine of the University of Giessen, Germany)
7. Michela Martini (Migration Health Regional Specialist, IOM Regional Office for Horn, East and Southern Africa, Nairobi, Kenya)
8. Moeketsi Modisenyane, National Department of Health, South Africa
9. Davide Mosca (Director, Migration Health Division, IOM, Geneva, Switzerland)
10. Kevin Pottie (Associate Professor, Faculty of Medicine, University of Ottawa, Ottawa, Ontario)
11. Bayard Roberts (Director, The Centre for Health and Social Change at the London School of Hygiene and Tropical Medicine, London, United Kingdom)
12. William Courtland Robinson (Associate Professor, Johns Hopkins Bloomberg, School of Public Health, USA)
13. Chesmal Siriwardhana (Associate Professor, London School for Hygiene and Tropical Medicine)
14. Ursula Trummer (Head, Center for Health and Migration, Vienna, Austria)
15. Jo Vearey (Associate Professor, African Centre for Migration & Society (ACMS), University of the Witwatersrand)
16. Kolitha Wickramage (Migration Health and Epidemiology Coordinator, IOM, Manila, Philippines)
17. Anthony Zwi (Professor of Global Health and Development, The University of New South Wales, Sydney, Australia)

## Abbreviations

ASEAN: Association of South-East Asian Nations
IOM: International Organization for Migration, the UN Migration Agency
MBDS: Mekong Basin Disease Surveillance
MHADRI: Migration, Health, and Development Research Initiative
SDGs: Sustainable Development Goals
WHO: World Health Organization
